# Co-design in action: lessons learned from transplant patient and family engagement in an integrated knowledge translation strategy during the COVID-19 pandemic

**DOI:** 10.1186/s40900-026-00941-1

**Published:** 2026-07-23

**Authors:** Margherita Cameranesi, Sherrie Logan, Rienk de Vries, Manuel Escoto, Marie-Josée Hébert, Dima Kabbani, Caroline Piotrowski, Lori West, Patricia Gongal

**Affiliations:** 1 Saint Mary’s University, Halifax, NS Canada; 2 Canadian Donation and Transplantation Research Program, Edmonton, AB Canada; 3 University of Alberta, Edmonton, AB Canada; 4 University of Manitoba, Winnipeg, MB Canada; 5 Université de Montréal, Montreal, QC Canada; 6 6002 Li Ka Shing Centre for Health Research Innovation, University of Alberta, Edmonton, AB T6G 2E1 Canada

**Keywords:** Community health, COVID-19, Healthcare policy, Integrated knowledge translation, Knowledge mobilization, Mental health, Organ donation, Organ transplantation, Patient engagement, Patient-oriented research, Patient and public involvement, Health equity

## Abstract

**Background:**

The COVID-19 pandemic significantly impacted immunocompromised individuals, particularly transplant recipients. Despite their vulnerability, national- and international-level research often lacks direct input from this community. In response, the Canadian Donation and Transplantation Research Program (CDTRP) implemented an integrated knowledge translation (iKT) strategy that involved patient, family, and donor (PFD) partners as co-leads and decision makers throughout the research process.

**Methods:**

Beginning in 2022, CDTRP adapted a co-engagement model to identify research priorities and inform study design, conduct, and knowledge mobilization related to COVID-19 issues for transplant recipients. Diverse stakeholders, including researchers, clinicians, policymakers, trainees, transplant-focused organizations, and PFD partners, collectively co-developed the TREAT-COVID research project. In 2022–2023 CDTRP hosted a series of four national forums that were co-designed and co-facilitated by PFD co-leads, enabling shared decision making and iterative refinement of research priorities and strategies. This paper provides an overview of the iKT strategy implemented, including objectives, engagement processes, outputs, resulting study adaptations, and lessons learned.

**Results:**

Each forum generated new insights and actionable changes. Forum 1 identified research priorities, including clinical therapeutics, psychosocial needs, quality of life, economic burden, and recovery-related supports. Forum 2 emphasized mental health and support for transplant recipients and their caregivers. Forum 3 identified the distinct but complementary priorities of transplant recipients and their caregivers, such as clinical care and mental wellness, respectively. Forum 4 addressed barriers to recruiting study participants and refined communication strategies. These forums collectively shaped the TREAT-COVID research project by informing study priorities, data collection tools, recruitment strategies, and knowledge mobilization.

**Conclusions:**

This paper details the iKT strategy implemented by the CDTRP to engage diverse stakeholders in COVID-19-related research for transplant communities. Meaningful and structured patient and family involvement in national health research during a public health emergency was prioritized. By involving patient and family partners in governance, facilitation, study refinement, recruitment, and dissemination, CDTRP advanced a collaborative and equity-driven iKT model. The paper contributes an operational example of national-scale patient and family co-leadership in time-sensitive research.

## Abstract (plain language summary)

The COVID-19 pandemic has posed major risks for immunocompromised individuals, including transplant recipients, but their voices are often underrepresented in research. To bridge this gap, the Canadian Donation and Transplantation Research Program (CDTRP) worked with transplant patient and family partners to develop a national research project that included their priorities. Patient and family partners were involved from the beginning, helping to shape research questions, lead meetings, review materials, identify challenges, and decide how results would be shared. In 2022–2023, CDTRP held four national forums to gather information from the transplant community, each with distinct goals. In Forum 1, participants discussed urgent research questions about treatments and mental health challenges faced. In Forum 2, participants refined research priorities, emphasizing mental health and supports for patients and families. In Forum 3, patients and caregivers highlighted distinct needs. In Forum 4, participants explored why transplant recipients and their caregivers and family members might or might not join the study and how to improve participation. A transplant recipient and a caregiver of a pediatric transplant recipient co-led these activities. They helped design and run the forums, reviewed results, and co-led the national research study. The forums shaped the TREAT-COVID project by broadening its focus, with the research team using input to improve the design and conduct of the study and knowledge sharing. This approach is a strong example of how persons with lived experience can be involved as equal partners in research, which is especially important when their lives and health are directly affected.

## Background

The World Health Organization declared COVID-19 a pandemic and global health emergency in March 2020, with the emergency phase of the pandemic ending in May 2023. Although the acute emergency phase has ended, the pandemic has caused enduring clinical, psychosocial, and health-system consequences for immunocompromised populations, including transplant recipients and their families. In Canada, approximately 40,000 people live with a solid organ transplant or allogeneic hematopoietic cell transplant, making the transplant community a relatively small but clinically complex population with distinct needs during public health emergencies.

People with solid organ transplants and allogeneic hematopoietic cell transplants were disproportionately affected by COVID-19 because of elevated risks of severe infection, hospitalization, intensive care admission, and mortality [1–4]. Their vulnerability was further compounded by reduced vaccine effectiveness associated with immunosuppressive therapies and an ongoing elevated risk of breakthrough infections [2, 3, 5]. Beyond these clinical risks, the pandemic also affected transplant recipients and their families through reduced access to routine and specialized care, social isolation, disrupted routines, uncertainty about infection risk, and heightened anxiety [6–8]. Caregivers and family members also experienced increased psychosocial burden, although some reported benefits associated with virtual care, such as reduced travel demands and improved access to appointments [9]. 

While there is a growing body of evidence on the psychosocial impacts of the pandemic on this population [6], research that has genuinely and meaningfully included transplant recipients, family members, caregivers, and donors as partners in identifying research priorities and shaping study design remains limited [7, 8]. This gap is important because research conducted during public health emergencies must be both scientifically responsive and grounded in the priorities of those most affected. For transplant communities, this requires mechanisms that can rapidly integrate clinical, research, policy, and lived-experience expertise into study design and implementation.

## Integrated knowledge translation and patient and public involvement

Knowledge translation (KT) refers to processes that support the synthesis, dissemination, exchange, and application of research evidence to improve health practices, programs, services, and policies [10–12]. Integrated knowledge translation (iKT) extends this approach by involving knowledge users throughout the research process rather than only at the point of dissemination [13, 14]. In iKT, researchers and knowledge users may collaborate to define research questions, select methods, interpret findings, and mobilize results [13]. This approach is intended to enhance the relevance, usability, and uptake of research by ensuring that those who will use or be affected by the findings contribute directly to the research process [14–16]. 

In this paper, we use ‘patient and public involvement’ (PPI) to refer to the meaningful involvement of people with lived experience and other relevant public contributors in the design, conduct, interpretation, and dissemination of research. Patient, family, and donor (PFD) partners refer specifically to transplant recipients, family members, caregivers, living donors, deceased donor family members, and others with lived experience of donation or transplantation who contribute as research partners rather than as study participants. The term PFD was coined by a group of Canadian Donation and Transplantation Research Program (CDTRP) PFD partners to better reflect broader lived experiences of those who do not see themselves as patients, such as family members and living donors. We use the term “transplant community” to refer broadly to transplant recipients, patients awaiting transplant, living donors, donor families, caregivers and family of transplant recipients, clinicians, researchers, and other stakeholders involved in donation and transplantation research, care, and policy. Accordingly, in this manuscript, “public” does not refer to the general population, but to PFD partners and other non-academic contributors whose lived experience and stakeholder perspectives informed the research process.

Although KT, iKT, and PPI are overlapping concepts, they are not interchangeable. KT describes a broad set of activities designed to support the use of research evidence; iKT emphasizes collaboration with knowledge users throughout the research process; and PPI foregrounds the involvement of patients, families, donors, caregivers, and public contributors as partners in research rather than passive participants. Together, these approaches provided the conceptual foundation for the iKT strategy described in this paper.

## Canadian donation and transplantation research program and the TREAT-COVID initiative

CDTRP was created in 2013 to bring together Canada’s diverse donation and transplantation research communities [17]. CDTRP integrates expertise across the health, natural, and social sciences, and facilitates collaboration between academic partners, clinicians, and the cell, tissue, and organ donor and transplant community. Operating a national research network, CDTRP provides infrastructure, methodological, and operational support for research and opportunities for collaboration, enabling investigators to develop research ideas, build partnerships, and strengthen the quality, relevance, and impact of their work. The network has grown to include more than 400 researchers, trainees, and knowledge users, including over 100 PFD partners, at 37 sites across Canada. A core feature of CDTRP is its commitment to PFD engagement and consensus-based approaches to identifying research priorities.

During the COVID-19 pandemic in Canada, public health measures and approaches to clinical care varied across provinces, and physicians often treated transplant recipients with limited evidence and rapidly changing guidelines. These persistent gaps prompted members of the CDTRP network to call for a coordinated, national research strategy to better prevent severe COVID-19 in transplant recipients, support rehabilitation and recovery, and establish mechanisms for shared data collection and use. The urgent and rapidly evolving nature of the pandemic demanded an agile research approach capable of responding to changing clinical evidence, public health guidance, and the priorities of transplant recipients and their families. In response, the CDTRP research team implemented an iKT strategy that engaged PFD partners as collaborators and decision makers throughout the research process. Together, a national network of researchers, clinicians, policymakers, and PFD partners co-created the TREAT-COVID research project to identify and respond to the research priorities of the transplant community during the COVID-19 pandemic.

## Rationale and contribution

This paper describes how the CDTRP, with the co-leadership of two PFD partners, took a comprehensive approach to PPI during a public health emergency. This multi-pronged approach included co-developing and delivering four national forums to identify research priorities, foster inclusive dialogue, and ensure that the lived experiences and priorities of transplant recipients, caregivers, and family members directly informed the design and implementation of the TREAT-COVID research project. We outline how the forums were structured, who was involved, what was learned, and how these lessons may inform future large-scale PPI efforts in complex, time-sensitive research environments.

The primary contribution of this paper is methodological and practice-oriented: we describe an operational model for national-scale PFD co-leadership in iKT during a public health emergency. By documenting how PFD partners were involved in governance, agenda-setting, facilitation, interpretation, and dissemination activities, this paper contributes to the literature on how patient-oriented research can be organized at scale when timelines are compressed, evidence is rapidly evolving, and affected communities face heightened clinical and psychosocial risk. We also reflect on the facilitators and challenges encountered throughout this work and discuss how these insights can inform future iKT strategies during public health emergencies.

## Methods

### Design and approach

We developed and implemented a national iKT strategy to co-design and refine the TREAT-COVID research project. The strategy was implemented by the CDTRP between March 2022 and December 2023 and centered on four national forums with transplant recipients, family members, caregivers, donors, clinicians, researchers, policymakers, trainees, CDTRP staff, and representatives of transplant-focused organizations.

The purpose of the forums was not to generate generalizable empirical findings, but to support shared priority setting, stakeholder engagement, study refinement, and knowledge exchange during a rapidly evolving public health emergency. Accordingly, this manuscript is presented as a descriptive iKT case example documenting how PFD co-leadership and PPI was operationalized in a national transplant research initiative. This section describes the governance structure, PFD involvement, recruitment and participation processes, forum procedures, and approach used to synthesize forum outputs. Forum-specific outputs and lessons learned are reported in the Results section.

### Ethical oversight

Ethics approval for the TREAT-COVID research project, including the forums, was obtained from the University of Alberta Research Ethics Board (Pro00131530). The forums were public events and open to all. Attendees were informed about the purpose of each forum and how information generated during the discussions would be summarized and used to inform the TREAT-COVID project. As the forums constituted community engagement events rather than research and attendees were not research participants, informed consent from attendees was not required nor sought. No individually identifiable quotations are reported in this manuscript. Demographic data were not systematically collected from forum participants. Therefore, participant characteristics are reported only in aggregate based on attendance information and the authors’ knowledge of the community of attendees.

### Governance and patient, family, and donor partner involvement

PFD involvement is documented using the Guidance for Reporting Involvement of Patients and the Public 2-Short Form checklist (Table 1). A collaborative governance structure was established to operationalize iKT principles across the project. The Steering Committee included two PFD co-leads, lead investigators, and research staff. The committee was responsible for aligning research priorities, study design, data collection tools, implementation planning, KT activities, and resource allocation. The two PFD co-leads, a pediatric transplant recipient caregiver (SL) and an adult transplant recipient (RdV), served as equal members of the leadership team. They contributed to priority setting, forum planning, facilitation, interpretation of forum outputs, refinement of study materials, recruitment and communication strategies, knowledge translation activities, and grant and manuscript development.

**Table 1:** Guidance for reporting involvement of patients and public (GRIPP) 2-short form

1. Aim: Report the aim of PPI* in the study We aimed to design the TREAT-COVID research study with PFDs to address the urgent and prioritized concerns raised by transplant recipients and their families and caregivers. The aim for integrating PFD partners into the study was to empower their perspective, which played a key role in shaping the study’s focus to ensure it directly reflected the real-world needs of the transplant community. Additional aims for integrating PFD partners included collaborating in the development of strategies for a more agility and responsive research framework that could better address emerging needs during the ongoing and evolving pandemic. Outreach to diverse patient and family organizations aimed to engage marginalized groups.
***2. Methods: Provide a clear description of the methods used for PPI in the study*** A structured, multi-stage patient and public involvement (PPI) approach was embedded throughout the study. PPI activities began with priority setting during the March 2022 forum, where PFD members identified key research questions and areas of concern. Two PFD co-leads—one a pediatric caregiver and the other an adult transplant recipient, were engaged immediately after this first forum, and held leadership roles across governance, study design, and implementation. As integral members of the Steering Committee, the PFD co-leads contributed to the development of the research questions, methodology, survey instruments, and the implementation of the study’s data platform. The PFD co-leads played a key role in KT by co-authoring academic manuscripts, including this one, and by helping develop plain language summaries to communicate findings to non-academic audiences. They served as co-applicants on numerous funding proposals. The PFD co-leads co-designed and co-facilitated two dedicated PFD Forums (June 2022 and June 2023) to refine research priorities, to ensure that the evolving needs of the transplant community remained central to the research agenda. During the research project implementation, a PFD Advisory group provided real-time input on study rollout. Members helped co-develop recruitment and retention strategies, with additional planning sessions integrated into the national forums. Advisory group members piloted study surveys to assess clarity, relevance, and plain language use, and reviewed study updates sent to participants, leading to refinements that improved accessibility for a broad and diverse population.
***3. Study results: Report the results of PPI in the study, including both positive and negative outcomes*** PPI activities led to several key positive outcomes that significantly enhanced the quality and relevance of the TREAT-COVID study. Research priorities and questions were defined by patients, caregivers, and donor partners, and were refined iteratively to reflect emerging community needs. Recruitment and communication strategies were co-developed with a focus on accessibility and inclusivity, ensuring that outreach efforts resonated with diverse audiences. Surveys were improved based on feedback from PFD members to enhance clarity, relevance, and language accessibility. PPI contributions also played a vital role in the success of funding applications and resulted in meaningful co-authorship (including co-first authorship) on academic outputs. In addition, KT materials were developed collaboratively, helping to ensure that research findings were disseminated in formats that were both understandable and useful to non-academic audiences. PPI activities also revealed some challenges and limitations. The absence of a formal Terms of Reference during the early stages of the project led to uncertainty about the roles, responsibilities, and decision-making authority of PPI contributors, which at times affected collaboration and clarity. Furthermore, some initial public-facing materials were developed without input from PPI members, requiring later revisions to ensure plain language and accessibility. These revisions delayed the study launch. These issues highlight the importance of clearly defined PPI structures and early, sustained involvement to support effective and equitable engagement.
***4. Discussion and conclusions: Comment on the extent to which PPI influenced the study overall. Describe positive and negative effects*** Embedding PPI throughout the study had several positive effects that strengthened its overall design and implementation. The involvement of PFD partners enhanced the study’s ethical integrity by ensuring that patient and caregiver voices were meaningfully included at every stage. Their lived experience contributed critical insights that grounded the research in real-world concerns, making the project more relevant to the transplant community. In addition, participant recruitment and retention strategies were improved through co-designed messaging and outreach approaches that reflected community expectations and values, thereby increasing trust and engagement. Despite these benefits, there were also limitations to the extent and inclusivity of PPI. Scheduling constraints and reliance on digital platforms for engagement may have unintentionally excluded individuals with limited access to technology or flexible time. As a result, the diversity of voices involved in PPI may not have fully reflected the broader national transplant community, particularly among underserved or marginalized groups. These challenges suggest that additional strategies, such as flexible engagement formats, targeted outreach, and resource supports, are necessary to make PPI more inclusive and representative in future studies.
***5. Reflections/critical perspective: Comment critically on the study***,*** reflecting on the things that went well and those that did not***,*** so others can learn from this experience*** Several key factors enabled effective patient and public involvement (PPI) in this study. Early and sustained engagement of PFD partners ensured that their perspectives shaped the project from inception through to dissemination. The inclusion of lived-experience experts in leadership roles contributed to a culture of shared ownership and mutual respect, while structured forums provided multiple opportunities for collaboration and iterative input. At the same time, the study also highlighted areas for growth and improvement in future PPI efforts. Expanding the diversity of PPI contributors is essential to ensuring that research reflects the full spectrum of patient experiences. Additionally, more formal training and capacity-building opportunities for both researchers and PFD members could enhance mutual understanding, improve communication, and strengthen the quality of engagement. These lessons underscore the importance of resourcing and structuring PPI as a core element of research, not as a peripheral activity. Clearly defined roles and responsibilities may help to support meaningful participation and decision-making, reinforcing the credibility and relevance of the research among both patient/family communities and academic/clinical stakeholders.

In addition to the PFD co-leads, members of a broader PFD advisory group contributed as advisors and knowledge mobilizers. They reviewed study materials for accessibility and relevance, supported the development of plain language summaries and dissemination tools, and helped identify barriers to recruitment in the TREAT-COVID study.

Further, the broader CDTRP PFD membership contributed through the national forums and CDTRP’s Mental Health Hub. These complementary structures created multiple pathways for PFD partners to influence the study. The Mental Health Hub was the space in which the PFD co-leads were identified and an early desire of the PFD community to incorporate this topic in the project was expressed.

The PFD co-leads and advisory committee members were offered an hourly rate of compensation to recognize the value of their time spent on the project, in line with the policies of the CDTRP PFD Platform.

**Fig. 1:**
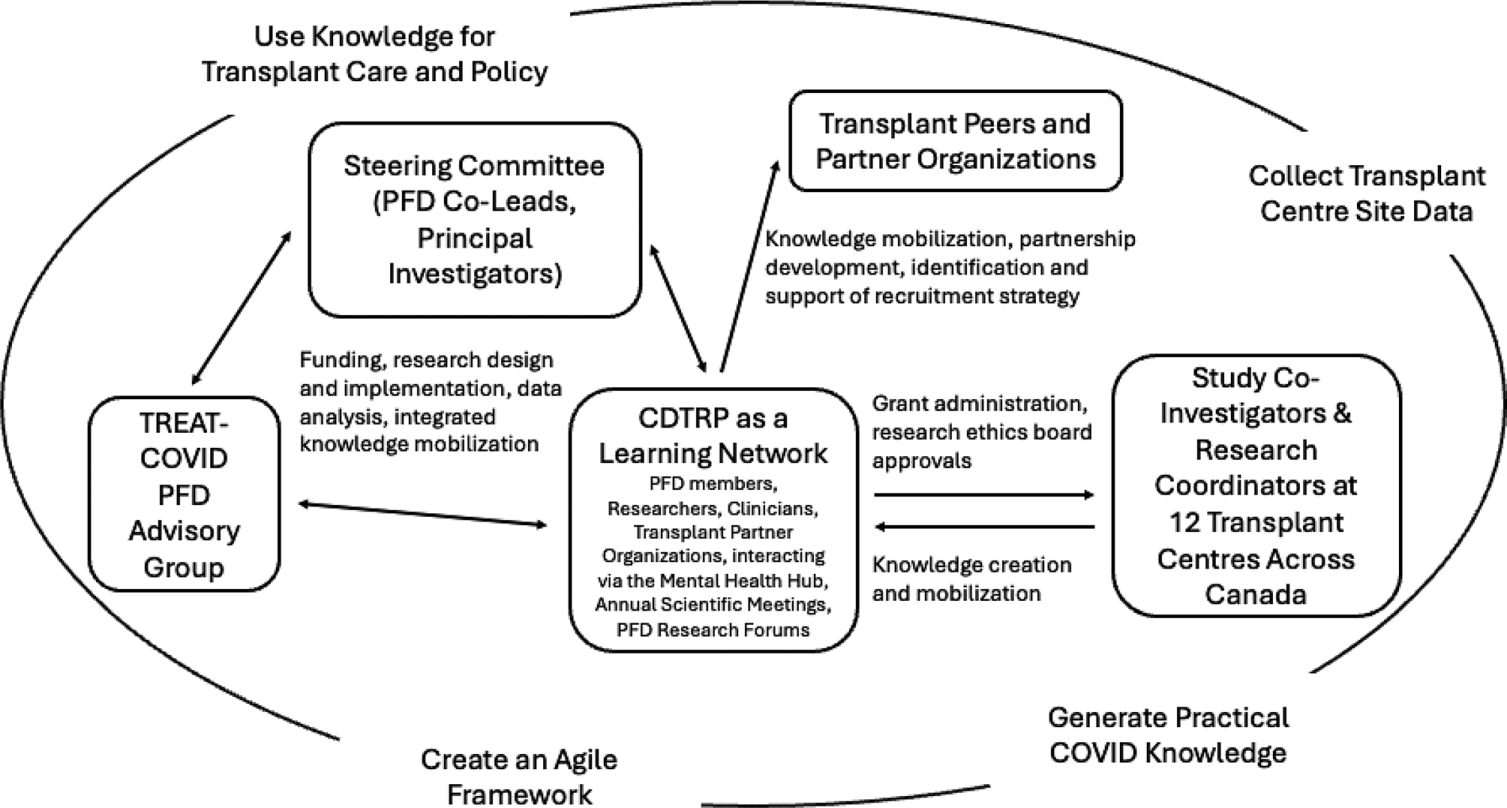
TREAT-COVID collaborative research framework

Figure 1 illustrates the collaborative research framework used to operationalize the TREAT-COVID iKT strategy. CDTRP functioned as a central learning network connecting PFD partners, researchers, clinicians, transplant partner organizations, the Steering Committee, the PFD Advisory Group, and study teams at 12 transplant centers across Canada. The bidirectional arrows represent reciprocal knowledge exchange across groups, including priority setting, study implementation, recruitment, data collection, knowledge mobilization (KM), and use of emerging evidence to inform transplant care and policy.

### Development of the TREAT-COVID iKT strategy

In early 2022, CDTRP leadership initiated the development of a national iKT strategy in response to the rapidly changing needs of transplant recipients during the COVID-19 pandemic. The strategy built on existing CDTRP partnerships and previous COVID-19 research with transplant recipients [5, 18–24]. CDTRP engaged a diverse network of stakeholders, including clinicians, researchers, policy advisors, and PFD partners, in a collaborative planning process.

Rather than relying on a one-time consultation model, the iKT strategy used a series of four national forums to iteratively identify priorities, refine study questions, identify recruitment barriers, and strengthen communication and dissemination strategies. The first two forums, held in March and June 2022, focused on defining research priorities and framing the study scope. After the TREAT-COVID project was funded, the third and fourth forums, held in June and December 2023, focused on project updates, alignment with evolving community needs, recruitment barriers, communication strategies, and implementation considerations.

### Forum promotion and participants

Forums were promoted through CDTRP’s national network, which includes researchers, clinicians, trainees, research staff, PFD partners, transplant-focused organizations, and other knowledge users. Promotional strategies included direct email invitations, email blasts to the CDTRP membership mailing list, social media posts from CDTRP and partners, and notices in partner organization newsletters.

Forum 1 was a stand-alone event. Forums 2 and 3 were integrated into CDTRP’s annual Patient, Family, and Donor Research Forum and Forum 4 was held alongside CDTRP’s national Annual Scientific Meeting. Forums 1, 2, and 3 were held virtually. Forum 4 offered both virtual and in-person participation options. All forums were conducted in English, and Forum 4 included a French session through a dedicated breakout group.

Because demographic and health data were not collected, we cannot report detailed participant characteristics such as age, gender, race/ethnicity, socioeconomic circumstances, transplant type, donation type, or time since transplant/donation. Forum objectives, participant categories, formats, and engagement methods are summarized in Table 2.

**Table 2:** Overview of national forums, participants, and engagement methods

	Forum 1	Forum 2	Forum 3	Forum 4
Date	March 2022	June 2022	June 2023	December 2023
Format	Virtual	Virtual	Virtual	Hybrid: in-person and virtual
Setting	Held as a stand-alone national forum convened by CDTRP to initiate pandemic-focused research priority setting	Integrated into CDTRP’s annual Patient, Family, and Donor Research Forum	Integrated into CDTRP’s annual Patient, Family, and Donor Research Forum	Held in conjunction with CDTRP’s national Annual Scientific Meeting
Main objective	Formulate and prioritize initial pandemic-focused research questions relevant to transplantation	Obtain feedback from transplant recipients, caregivers, and families on the psychosocial and economic impacts of the pandemic	Assess whether TREAT-COVID research goals remained aligned with the evolving priorities of transplant recipients, caregivers, and families	Identify and address early barriers to TREAT-COVID recruitment, participation, and communication
Participants	30 participants including clinicians, researchers, transplant recipients, pharmacists, industry, government health policy analysts, health economist	25 participants including patient, family, and donor partners, clinicians, researchers and trainees	25 participants including patient, family, donor partners, trainees, clinicians, researchers	60 patient, family, and donor partners, researchers, clinicians, trainees, and representatives of transplant-focused organizations
Engagement methods	2-hour virtual meeting, including 45 min of breakout discussion, with groups based on self-selection by interest	90-minute virtual meeting, including polls, virtual whiteboard for brainstorming, and 20 min of breakout discussion, with groups divided into transplant recipients and caregivers	90-minute virtual meeting, with 40 min of breakout discussions, with groups divided into transplant recipients and caregivers	90-minute hybrid event, including 30 min of multi-lingual in-person and virtual breakout discussions. Forum 4 also included a French-language breakout option
Primary output	Three initial research priorities: COVID-19 therapeutics, psychosocial and quality-of-life impacts, and supports needed for recovery and return to daily life	Refined understanding of psychosocial priorities, including information overload, isolation, family strain, policy gaps, anxiety, depression, peer support needs, and family-focused education needs	Identification of distinct patient and caregiver priorities, with transplant recipients emphasizing clinical treatment questions and caregivers emphasizing mental health and wellness	Identification of recruitment and communication barriers, motivators for participation, and strategies for trusted, community-based outreach

### Forum design and procedures

Each forum was designed to support structured engagement, shared priority setting, and iterative refinement of the TREAT-COVID project, based on the status of the pandemic and the project at the time. PFD partners were involved in forum planning, facilitation, and interpretation, with the level and form of involvement varying across forums. Forum procedures are summarized in Table 3.

**Table 3:** Forum outputs, resulting study adaptations, and PFD contributions

Forum	Key Outputs	Resulting Study Adaptation	PFD Contribution
Forum 1	Three priority areas were identified: (1) COVID-19 therapeutics, including antiviral agents and monoclonal antibodies; (2) psychosocial impacts, mental health, quality of life, and economic burden; and (3) supports needed to help transplant recipients and families return to daily life. Participants also identified the need for better coordination across sectors and clearer, more timely communication about evolving COVID-19 guidance.	The TREAT-COVID research agenda was broadened from an initial emphasis on clinical therapeutics to include mental health, quality of life, economic burden, recovery, and return-to-life concerns. The research team was expanded, including integration of PFD co-leadership.	PFD participants helped shift the research agenda beyond clinical treatment questions by emphasizing mental health, psychosocial burden, communication gaps, stigma, and the everyday realities of living as an immunocompromised person during the pandemic.
Forum 2	Participants identified key pandemic-related challenges, including information overload, social isolation, policy gaps, family strain, anxiety, depression, and unmet needs for peer support, family-focused education, and public understanding of immunocompromised populations.	Findings from Forum 2 informed the development of TREAT-COVID priorities related to psychosocial impacts, mental health supports, family/caregiver needs, and economic burden, alongside clinical questions about therapeutic safety and effectiveness.	PFD co-leads planned, promoted, and co-chaired the forum; developed engagement activities; facilitated breakout discussions; and helped interpret the psychosocial priorities raised by transplant recipients and caregivers.
Forum 3	Distinct priorities emerged across participant groups: transplant recipients focused primarily on clinical treatment questions, whereas caregivers emphasized mental health, wellness, and family-related impacts. Participants also raised concerns about the limited socioeconomic diversity represented in forum discussions.	Study materials and data collection tools were refined to better capture caregiver-specific experiences, mental health indicators, stigma-related concerns, and the potentially different priorities of transplant recipients and caregivers. The need for more inclusive outreach was also identified.	PFD co-leads summarized findings from earlier forums, co-facilitated discussion, supported psychologically safe participation, and helped identify the importance of treating patient and caregiver experiences as related but distinct knowledge domains.
Forum 4	Participants generated recommendations to address recruitment and communication barriers, including questionnaire burden, pandemic fatigue, limited perceived short-term benefit, and the need for trusted community-based messengers. Participants also recommended accessible formats, plain language, culturally relevant communication, and outreach through peer/community networks.	Recruitment and communication strategies were revised to emphasize study relevance, community benefit, future emergency preparedness, trusted messengers, plain language, accessible formats, and decentralized communication through credible community organizations.	PFD partners identified practical barriers to participation, recommended more accessible and community-centered messaging, and positioned patients, caregivers, families, and donors as active co-implementers of recruitment and dissemination strategies.

Note. PFD = patient, family, and donor. Forum outputs were synthesized descriptively from forum discussions, polling, virtual whiteboard activities, facilitator notes, and full-group reflections

Forum 1 was held online in March 2022 and focused on identifying pandemic-related research priorities for the transplant community. Participants discussed four preliminary areas: the use of antiviral agents, the use of monoclonal antibodies, mechanisms to support rapid awareness of emerging therapeutic agents, and issues related to recovery and long-term well-being for patients and family members. Participants joined virtual breakout groups based on expertise and interest. Breakout discussions were documented using shared online notes, and participants reconvened to identify common issues and provide feedback on emerging priorities.

Forum 2 was held online in June 2022 and focused on refining psychosocial and economic priorities for transplant recipients and their families. The session was planned, promoted, and co-chaired by the PFD co-leads. Participants completed anonymous polling questions, contributed to a Google Jamboard activity, and joined breakout groups for transplant recipients and caregivers. The session concluded with a large-group discussion to review and validate key themes generated during the forum.

Forum 3 was held online in June 2023 and focused on providing a TREAT-COVID project update, identifying information needs among transplant patients and families, and assessing whether the research priorities remained aligned with evolving community needs. PFD co-leads summarized findings from previous forums and moderated breakout discussions. Participants used Google Jamboard to identify research questions related to COVID-19 treatment, mental health and wellness, and financial burden.

Forum 4 was held in December 2023 and focused on barriers to participation in the ongoing TREAT-COVID study, including questionnaire burden, study coordinator burden, and pandemic fatigue [25]. The forum included virtual and in-person participation. Participants joined facilitated breakout groups to discuss reasons transplant recipients might not enrol in the study, motivations for participation and retention, communication platforms and organizations that could support recruitment, and credible messengers for national outreach. Each breakout group included a trained facilitator and notetaker and at least one PFD contributor.

### Data sources and synthesis of forum outputs

Forum outputs were generated through multiple methods, including facilitator notes, shared online notes, Google Jamboard, anonymous polling, large-group discussion notes, and real-time participant validation. CDTRP staff and forum facilitators documented discussions during each forum. Where possible, participants were invited to review, clarify, or expand on emerging points during the sessions. After each forum, CDTRP staff and the PFD co-leads reviewed the notes and summarized key priorities, concerns, recommendations, and implications for the TREAT-COVID project. These summaries were used to refine study priorities, adapt recruitment and communication materials, inform survey development, and guide dissemination planning.

**Fig. 2:**
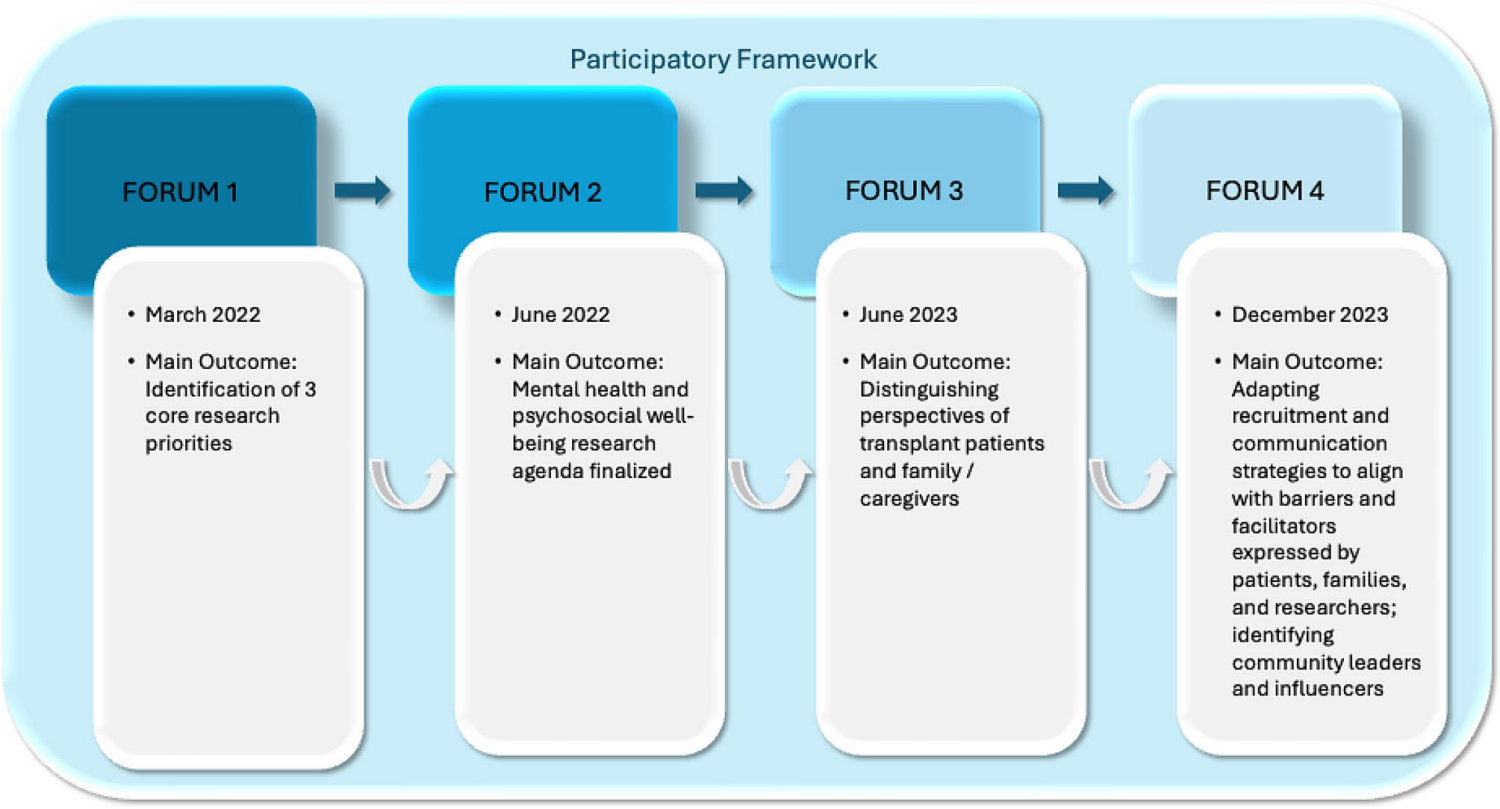
Outcomes of each forum of the implemented iKT strategy

Given the KT and engagement purpose of the forums, outputs were synthesized descriptively rather than analyzed as formal qualitative research data. The synthesis focused on identifying themes, actionable priorities, engagement lessons, and study adaptations arising from each forum. Forum-specific outputs are reported in the next section, and cross-forum lessons are summarized in Table 3; Fig. 2.

## Results

### Overview of forum participation and outputs

Across the four national forums to support the development, refinement, and implementation of the TREAT-COVID research project, 140 participants contributed to the iKT process, including transplant recipients, family members, caregivers, donors, clinicians, researchers, trainees, research staff, policymakers, industry representatives, and representatives from transplant-focused organizations. Attendance varied across forums: Forum 1 included 30 participants, Forum 2 included 25 participants, Forum 3 included 25 participants, and Forum 4 included 60 participants. Participants included PFD partners, clinicians, researchers, trainees, CDTRP staff, policymakers, transplant organization representatives, and other knowledge users.

Each forum generated outputs that informed subsequent stages of the TREAT-COVID project. Early forums focused on identifying and refining research priorities, whereas later forums focused on ensuring alignment with evolving community needs, improving recruitment, and strengthening communication strategies. Key forum outputs, resulting study adaptations, and PFD contributions are summarized in Table 3. Figure 2 provides a visual overview of the main outcomes generated across the four forums.

### Forum 1: Identifying initial research priorities

Forum 1 generated three core research priorities. First, participants identified the need to examine the optimal timing, safety, and effectiveness of COVID-19 therapeutics, including antiviral agents and monoclonal antibody therapies, for transplant recipients. Second, participants emphasized the importance of understanding the broader consequences of pandemic-related policies, including impacts on mental health, quality of life, economic burden, and the ability to safely return to daily life. Third, participants identified the need for supports within transplant centers and communities to assist transplant recipients and families in recovery and adaptation during and after the pandemic.

PFD input was central to broadening the initial research agenda. Although early discussions were primarily focused on clinical therapeutics, transplant recipients and family members emphasized that mental health, stigma, communication gaps, social isolation, and quality of life were also urgent research priorities. As a result, the emerging TREAT-COVID agenda expanded from a primarily biomedical focus to a broader patient-informed agenda that included psychosocial, economic, and recovery-related concerns. Following Forum 1, a member of the research team presented the project to the CDTRP Mental Health Hub to solicit participation to develop these components of the project. This resulted in the expansion of the research team and identification of the PFD co-leads.

Forum 1 also highlighted the need for improved coordination across healthcare, academic, government, industry, and community sectors. Participants noted that fragmented communication during the pandemic created barriers to timely and coordinated research responses.

### Forum 2: Refining psychosocial and economic priorities

Forum 2 further refined the psychosocial and economic priorities identified in Forum 1. Participants identified several pandemic-related challenges, including information overload, social isolation, policy gaps, family strain, anxiety, depression, and uncertainty about public health guidance for immunocompromised people. Polling and group discussion suggested that participants’ well-being had declined during key phases of the pandemic, particularly during the early months and subsequent COVID-19 waves.

Participants also identified sources of support, including family members, healthcare providers, transplant organizations, and media, while also noting unmet needs. These included peer support groups, family-focused education, clearer information tailored to immunocompromised populations, and greater public understanding of the ongoing risks faced by transplant recipients and their families.

The two PFD co-leads independently designed, facilitated, and interpreted Forum 2, strongly supporting the integration of lived-experience priorities into the study’s evolving design. Findings from Forum 2, together with Forum 1 outputs, informed the development of three PFD-informed TREAT-COVID project priorities: (1) evaluating the safety and effectiveness of COVID-19 prophylactic and therapeutic interventions; (2) assessing the psychosocial impacts of the pandemic and identifying strategies to improve well-being; and (3) examining the economic burden and value of available COVID-19 interventions.

### Forum 3: Reassessing priorities and distinguishing patient and caregiver needs

Forum 3 was designed to assess whether TREAT-COVID research goals continued to align with the evolving needs of transplant recipients and families. A key output of this forum was the identification of differences between transplant recipient and caregiver priorities. Transplant recipients focused primarily on clinical treatment questions, while caregivers emphasized mental health, wellness, and family-related impacts. This distinction informed refinements to TREAT-COVID data collection tools, including stronger attention to caregiver-specific experiences, mental health indicators, and stigma-related concerns. The forum also reinforced the importance of treating transplant recipients and caregivers as overlapping but distinct groups whose needs and priorities may differ.

Participants also raised concerns about the limited socioeconomic diversity represented in the forum discussions. This observation informed subsequent efforts to strengthen outreach to communities and individuals who may be less frequently engaged in research. Because detailed demographic and transplant-related characteristics were not systematically collected, the extent of diversity across participant groups could not be fully assessed.

### Forum 4: Addressing recruitment and communication barriers

Forum 4 focused on early barriers to recruitment and participation in the TREAT-COVID study. Participants generated 273 statements addressing reasons transplant recipients might not enrol, motivations for participation and retention, communication platforms and organizations that could support recruitment, and credible messengers for national outreach.

Key barriers included questionnaire burden, pandemic fatigue [25], limited perceived short-term benefit of participation, and the burden placed on study coordinators. Participants also identified motivators for involvement, including the opportunity to contribute to community well-being, improve future emergency preparedness, and ensure that transplant recipients’ experiences were represented in research.

Forum 4 outputs directly informed revisions to recruitment and communication strategies. These revisions included emphasizing the continued relevance of COVID-19 for transplant communities, communicating the potential value of the study for future emergency preparedness, using plain language and accessible formats, and disseminating information through trusted community-based messengers and transplant-focused organizations. PFD partners were central to identifying practical barriers to participation and reframing recruitment as a community-engaged process rather than a solely investigator-led activity.

### Cross-forum outputs and study adaptations

Across the four forums, several cross-cutting outputs emerged. First, PFD involvement broadened the research agenda beyond clinical therapeutics to include psychosocial well-being, quality of life, communication, stigma, family burden, economic concerns, and recovery needs. Second, the forums demonstrated that transplant recipients and caregivers may have distinct but complementary priorities, requiring separate attention in study design and data collection. Third, participants emphasized the importance of trusted, accessible, and community-based communication strategies in supporting recruitment and engagement.

The forums led to concrete adaptations to the TREAT-COVID project. These included adding entirely new research priorities, establishing a governance structure with PFD co-leads, strengthening caregiver-specific data collection, incorporating mental health and stigma-related components, and revising recruitment and communication materials to better reflect community priorities. Together, these outputs demonstrate how the national forums functioned as an iterative iKT mechanism through which lived experience and research expertise were brought together to shape a national transplant research initiative.

## Discussion

This paper describes the development and implementation of a national iKT strategy used to co-design and refine the TREAT-COVID research project during the COVID-19 pandemic. With PFDs in leadership and advisory roles, and broader engagement through national forums, CDTRP involved people with lived experience as well as the broad transplant community of clinicians, researchers, trainees, policymakers, industry representatives, and transplant-focused organizations. These diverse perspectives were integrated to identify research priorities, refine the study design, address recruitment barriers, and strengthen communication strategies. The primary contribution of this paper is methodological and practice-oriented: we document an operational model for national-scale PFD co-leadership in iKT during a rapidly evolving public health emergency.

Across the forums, several key lessons emerged. First, PFD involvement broadened the research agenda significantly, crossing research disciplines. Second, transplant recipients and caregivers identified related but distinct priorities, reinforcing the importance of engaging these groups separately as well as collectively. Third, recruitment and communication strategies were strengthened when PFD partners helped identify barriers, trusted messengers, and accessible formats. Finally, the CDTRP network provided an enabling infrastructure through which lived experience, clinical expertise, and research priorities could be brought together iteratively across multiple sites and stakeholder groups.

### Operationalizing national-scale PFD co-leadership

A core strength of this iKT strategy was the meaningful co-leadership of PFD partners across multiple stages of the TREAT-COVID project. Two PFD co-leads, a transplant recipient and a caregiver, joined the Steering Committee early in the process and contributed to forum planning, facilitation, interpretation of forum outputs, study refinement, recruitment strategies, dissemination, and manuscript development. Their involvement positioned PFD partners as collaborators and decision makers within the project’s governance structure.

This approach illustrates how PPI can be operationalized within iKT at a national scale. Rather than treating lived experience as a single point of input, the TREAT-COVID strategy created repeated opportunities for PFD partners to shape decisions as the pandemic context evolved. This iterative structure was particularly important in an emergency research context, where clinical evidence, public health guidance, participant concerns, and recruitment conditions changed over time. PFD co-leadership helped ensure that the research agenda remained responsive to community priorities while also supporting trust, relevance, and accessibility.

### Promoting inclusive participation and remaining equity gaps

Inclusive participation was a central aim of the TREAT-COVID iKT strategy. Across the forums, the project team used multiple engagement strategies, including real-time polling, virtual whiteboards, breakout discussions, shared notetaking, and opportunities for both verbal and written input. Forums were hosted primarily virtually, with Forum 4 offering hybrid participation and a French-language breakout option. These strategies were intended to reduce participation barriers and create multiple ways for participants to contribute.

However, important challenges to equity and inclusion remained. Forums 1 through 3 were conducted in English only, which may have limited engagement from non-English-speaking participants. Virtual formats may have improved geographic reach but also may have created barriers for individuals with limited internet access, lower digital literacy, competing work demands, or caregiving responsibilities. In addition, detailed demographic, socioeconomic, transplant-related, and donation-related characteristics were not systematically collected, limiting our ability to assess how representative forum participation was across the broader transplant community. Future iKT strategies could combine flexible engagement modalities with targeted recruitment, multilingual options, accessibility planning, and minimum participant-characteristic reporting where ethically and practically appropriate.

### Aligning research objectives with evolving stakeholder priorities

The forums demonstrated the value of revisiting research priorities over time. In Forum 1, the initial focus on COVID-19 therapeutics expanded after PFD participants emphasized psychosocial burden, mental health, quality of life, communication challenges, and return-to-life concerns. Forum 2 further refined these priorities by identifying information overload, social isolation, policy gaps, family strain, anxiety, depression, and unmet needs for peer support and family-focused education. Together, these early forums shaped a broader TREAT-COVID agenda that included therapeutic safety and effectiveness, psychosocial impacts, and economic burden.

Forum 3 further showed that transplant recipients and caregivers may hold different but complementary priorities. Transplant recipients emphasized clinical treatment questions, whereas caregivers emphasized mental health, wellness, and family-related impacts. This distinction informed refinements to study tools, including greater attention to caregiver-specific experiences, mental health indicators, and stigma-related concerns. These findings suggest that iKT strategies should avoid assuming that all members of a community with lived experience share the same priorities. Separate and combined engagement spaces may both be needed to identify convergent and divergent priorities across stakeholder groups.

### Producing actionable outputs for recruitment, communication, and KM

The iKT strategy produced practical outputs that informed the design and implementation of TREAT-COVID. Feedback from the forums shaped research questions, survey content, recruitment approaches, and knowledge translation products. PFD partners contributed to plain language summaries and dissemination materials, supporting the accessibility and relevance of study communication.

Forum 4 was particularly important in translating engagement into implementation strategies. Participants identified barriers to enrolment and retention, including questionnaire burden, pandemic fatigue, limited perceived short-term benefit, and study coordinator burden. They also identified motivators for participation, including the opportunity to contribute to the transplant community and improve future emergency preparedness. These insights led to revisions in recruitment and communication materials, including greater emphasis on study relevance, community benefit, trusted messengers, plain language, accessible formats, and outreach through transplant-focused and community-based organizations.

These outputs demonstrate that PPI-informed iKT can support more than priority setting. In this project, PFD partners also contributed to implementation, recruitment, communication, and KM. This is an important practice implication for future emergency research: persons with lived experience should not only be asked what research matters, but also how research can be communicated, implemented, and sustained in ways that are credible and accessible.

### Implications for future PPI-informed iKT strategies

The TREAT-COVID experience suggests several implications for future patient-oriented research in complex or time-sensitive contexts. First, PFD roles should be defined early and supported with appropriate resources, including time, compensation, preparation, and clear expectations. Second, engagement should be iterative rather than one-time, particularly when public health conditions and community priorities are changing rapidly. Third, national research networks can play an important convening role by linking PFD partners, researchers, clinicians, policymakers, community organizations, and local study sites. Fourth, recruitment and dissemination strategies should be co-developed with community partners who understand local barriers, trusted communication channels, and accessibility needs.

Finally, future iKT strategies should build participant-characteristic reporting into engagement activities when appropriate. Although engagement forums are not always designed as formal research data collection activities, collecting a small set of aggregate, non-identifying characteristics may help teams assess whose perspectives are represented and whose perspectives remain underrepresented. This is particularly important for equity-oriented research, where claims about inclusion should be supported by transparent reporting of participation and representation.

### Strengths and limitations

This work has several strengths. This national iKT strategy implemented during a public health emergency provides a concrete example of how PFD partners can contribute to governance, priority setting, forum facilitation, study refinement, recruitment, communication, and dissemination. The use of four forums over time allowed the research team to revisit priorities as the pandemic evolved, rather than relying on a single consultation. The involvement of PFD co-leads and a broader advisory structure also strengthened the relevance and responsiveness of the TREAT-COVID project.

Several limitations should also be considered. First, demographic, socioeconomic, transplant-related, donation-related, and time-since-transplant or time-since-donation information was not systematically collected from forum participants. Thus, we cannot determine where participants were in their donation or transplantation journey, nor can we fully assess the diversity or representativeness of forum participants. Second, most forums were conducted in English and online, which may have limited participation from non-English-speaking individuals and those with limited digital access. Third, although the forums generated actionable priorities and study adaptations, the impact of the iKT strategy on study recruitment, participant experience, policy uptake, or clinical outcomes was not formally evaluated. Fourth, because this paper reports a descriptive iKT case example from a specific national transplant research network, the transferability of lessons to other settings may depend on available infrastructure, existing relationships, and resources for patient-oriented research.

Despite these limitations, the TREAT-COVID experience offers a practical example of how national research networks can embed PFD co-leadership into time-sensitive research. The forums created structured opportunities for lived experience to shape what research questions were asked and how the study was designed, communicated, implemented, and mobilized. Future work should build on this model by evaluating the outcomes of PPI-informed iKT strategies and identifying the conditions under which they most effectively improve research relevance, equity, implementation, and impact.

## Conclusions

This manuscript describes a national PPI-informed iKT strategy used to co-design and refine the TREAT-COVID research project during the COVID-19 pandemic. Through four national forums, PFD partners contributed to priority setting, governance, forum facilitation, study refinement, recruitment planning, and knowledge mobilization. Their involvement helped broaden the research agenda beyond COVID-19 therapeutics to include mental health, caregiver experiences, communication needs, quality of life, and recovery-related priorities.

The TREAT-COVID experience illustrates how national research networks can embed PFD co-leadership into time-sensitive research while maintaining attention to relevance, responsiveness, and equity. It also highlights the need for early role clarity, accessible engagement strategies, intentional outreach to underrepresented communities, and infrastructure to support sustained collaboration. Future iKT initiatives, particularly during public health emergencies, should move beyond one-time consultation and create structured opportunities for patients, families, donors, clinicians, researchers, and decision makers to shape research as it evolves.

## Data Availability

The summary notes of the forums are publicly available online at: https://cdtrp.ca/en/publications/covid-19-vaccination-in-transplant-recipients-cdtrp-national-initiative/.
